# Increased Primary Production from an Exotic Invader Does Not Subsidize Native Rodents

**DOI:** 10.1371/journal.pone.0131564

**Published:** 2015-08-05

**Authors:** Jacob E. Lucero, Phil S. Allen, Brock R. McMillan

**Affiliations:** Department of Plant and Wildlife Sciences, Brigham Young University, Provo, Utah, United States of America; University of Naples 'Federico II', ITALY

## Abstract

Invasive plants have tremendous potential to enrich native food webs by subsidizing net primary productivity. Here, we explored how a potential food subsidy, seeds produced by the aggressive invader cheatgrass (*Bromus tectorum*), is utilized by an important guild of native consumers – granivorous small mammals – in the Great Basin Desert, USA. In a series of field experiments we examined 1) how cheatgrass invasion affects the density and biomass of seed rain at the ecosystem-level; 2) how seed resources from cheatgrass numerically affect granivorous small mammals; and 3) how the food preferences of native granivores might mediate the trophic integration of cheatgrass seeds. Relative to native productivity, cheatgrass invasion increased the density and biomass of seed rain by over 2000% (*P* < 0.01) and 3500% (*P* < 0.01), respectively. However, granivorous small mammals in native communities showed no positive response in abundance, richness, or diversity to experimental additions of cheatgrass seeds over one year. This lack of response correlated with a distinct preference for seeds from native grasses over seeds from cheatgrass. Our experiments demonstrate that increased primary productivity associated with exotic plant invasions may not necessarily subsidize consumers at higher trophic levels. In this context, cheatgrass invasion could disrupt native food webs by providing less-preferred resources that fail to enrich higher trophic levels.

## Introduction

Invasive plants have tremendous potential to influence the amount of resources available to higher consumers because of their generally positive effect on primary production and nutrient availability [1–2]. In a recent meta-analysis, Liao et al. [1] reported that invaded habitats yielded on average 83% greater net primary productivity than non-invaded habitats. In addition, plant invasions can also increase ecosystem nutrient availability, which often results in positive feedback to primary production [1–3]. This enhanced productivity and nutrient availability could broaden the base of resources available to higher consumers in invaded systems and potentially subsidize native food webs. However, whether increased productivity associated with plant invasion actually fluxes through native food webs is controversial.

Increased productivity from invasive plants may or may not subsidize native food webs. For example, seed heads from spotted knapweed (*Centaurea stoebe*), an aggressive invader in North America, support the larvae of *Urophora* gall flies, specialists introduced as biological controls. In turn, the larvae help sustain many native consumers, including deer mice (*Peromyscus maniculatus*) [4–5]. Additional food supplied by gall fly larvae in knapweed-invaded habitat can increase mouse populations up to 200% [5]. Thus, food subsidies provided by invasive knapweed are valuable to at least two trophic levels of consumers (gall flies and deer mice), and possibly a third (tertiary consumers feeding on deer mice). Alternatively, however, exotic resources may not subsidize native consumers. Although deer mice readily harvest *Urophora* larvae, they strongly avoid the seeds of spotted knapweed, showing a distinct preference for native species of Asteraceae in choice tests [6]. Rodent exclusion in field experiments increased the recruitment of preferred native species but not spotted knapweed. Thus, seed predation by deer mice limited the recruitment of native species but not knapweed. The failure of deer mice to consume enough spotted knapweed seeds to limit its recruitment suggests an alternative trophic scenario in which food resources from a dominant invader do not necessarily flux to local consumers.

Researchers could directly assess whether resources supplied by exotic invasions subsidize native food webs via experimental additions of exotic resources. If productivity from plant invasions subsidizes local food webs, supplemented consumers should respond positively to resource additions relative to unsupplemented controls. Positive responses could include increased consumer abundance, species richness, or diversity. Ideally, supplementation treatments would occur in the field at multiple sites representing spatially-independent consumer communities and would span multiple consumer generations. Interestingly, the potential for plant invasions to subsidize native food webs has rarely been evaluated in this way.

The Great Basin Desert, USA is an excellent system in which to experimentally assess whether plant invasion enriches native food webs. Much of the Great Basin was dominated by perennial grasses and shrubs prior to European settlement [7]. However, cheatgrass (*Bromus tectorum*) invasion has converted millions of hectares of Great Basin shrub-steppe to annual grasslands since the late 19^th^ century [7]. Cheatgrass invasion alters fire regimes [8], nutrient cycling [9–10], and habitat architecture [11] to the detriment of native species, and is considered one of the most significant plant invasions in North America [12]. Despite these disruptions, however, primary productivity (i.e., food resources) supplied by cheatgrass could potentially benefit native consumers by subsidizing their diets. Cheatgrass covers the landscape with dense, herbaceous biomass [13], making some cheatgrass-invaded grasslands more productive than native grasslands [14]. Also, cheatgrass is a prolific seed producer [15] and forms denser seed banks than adjacent, native species [16]. There is some evidence that native folivores including grasshoppers (e.g., *Xanthippus corallipes*, *Melanoplus confuses*) [17] and elk (*Cervus canadensis*) [18] could benefit from increased herbaceous productivity, and native granivores like pocket mice (*Perognathus parvus*) and deer mice might benefit from increased seed productivity [19–21].

However, the extent to which cheatgrass is actually integrated into native food webs is unknown. Assimilation is likely affected by the food preferences of native consumers. If exotic resources are preferred by native consumers [22], their assimilation and flux to higher trophic levels may be more likely than if exotic resources are avoided by native consumers [6, 23–24]. Importantly, native consumers in the Great Basin appear to avoid cheatgrass (e.g., [25]). Although some native herbivores do consume cheatgrass under certain conditions [18], foliage from native grasses appears to be preferred [26]. Similarly, despite the very high abundance of cheatgrass seeds in invaded habitats [15–16], native granivores often prefer native seeds [25, 27–29]. Notably, intensive cheatgrass invasion corresponds with sharp decreases in the abundance of native granivorous small mammals [30–32], possibly due to degradation of habitat structure [11].

Many studies have documented ecological disruptions caused by invasive plants, including their strong effects on resource and energy pools [1–2], but few have linked these ecosystem effects to their trophic consequences. Here, we explored the potential for seeds produced by the aggressive invader cheatgrass to subsidize an important guild of native consumers—granivorous small mammals—in the Great Basin Desert, USA. In a series of field experiments we examined 1) how cheatgrass invasion affected the density and biomass of seed rain at the ecosystem-level; 2) how small mammal communities numerically responded to experimental additions of cheatgrass seeds over one year; and 3) how the food preferences of these granivores related to their response to cheatgrass supplementation.

## Methods

### Study sites

We conducted our field experiments at various, spatially-independent plant communities in Rush Valley, Tooele County, Utah, USA. We conducted all experiments on public land managed by the Bureau of Land Management (BLM). Relevant permits were obtained through the Salt Lake City Field Office of the United States BLM, Salt Lake City, Utah, USA.

Rush Valley is characterized by a mosaic of near-monocultures of cheatgrass adjacent to intact, native communities dominated by perennial shrubs and bunchgrasses. Dominant shrubs include big sagebrush (*Artemisia tridentata*), black greasewood (*Sarcobatus vermiculatus*), and black sagebrush (*Artemisia nova*). Dominant bunchgrasses include Sandberg bluegrass (*Poa secunda*), Indian ricegrass (*Achnatherum hymenoides*), and bottlebrush squirreltail (*Elymus elymoides*). Native communities in Rush Valley are typified by relatively barren interspaces between shrubs with very few invasive species. In contrast, shrub interspaces in invaded communities are filled by cheatgrass that has displaced natives.

### Cheatgrass invasion and seed resources

We evaluated the effect of cheatgrass invasion on primary production in the form of seeds by comparing the density and biomass of seed rain between cheatgrass-invaded and non-invaded habitat. We measured seed rain on 3 transects in cheatgrass-invaded (hereafter “invaded”) and 3 transects in cheatgrass-noninvaded (hereafter “non-invaded”) communities of big sagebrush. Transect locations were selected to represent typical invaded and non-invaded communities in our study area. All transects were separated by >10 km and were thus spatially independent from each other. Invaded transects were characterized by 50–75% cheatgrass cover in shrub interspaces while non-invaded transects consisted of 0–5% cheatgrass cover (see [33] for protocol). Each transect was 110 m and consisted of 12 sampling stations spaced 10 m apart. We placed two seed traps at each station (24 seed traps total per transect)–one directly underneath the canopy of the nearest living shrub, and the other in open space ≥50 cm from the canopy of the nearest living shrub. We thus sampled seed productivity from shrubs, grasses, and forbs both under shrubs and in the open.

Seed traps consisted of plastic funnels (6.7 cm-diameter rim tapering to a 1.2 cm-diameter stem) fixed to 118.3 mL specimen jars. We drilled two drainage holes into the bottom of each jar. We buried seed traps with their rims 3–5 mm above the soil surface (see Fig 1 in [34]). We installed traps 18–23 Dec 2009 and left them in place until 18 Nov 2010. We collected seed rain data on 13 April 2010, 6 July 2010, 25 Aug 2010, and 18 Nov 2010. After collection, we immediately placed samples in a freezer for storage until sorting and analysis.

We thawed and dried the samples in an oven at 60°C for 12 hours and then separated seeds from other organic debris with tweezers under a dissecting microscope, determining viability via the firmness of seeds following Price and Joyner [34]. We counted and weighed only viable seeds to calculate the density (seeds/m^2^) and biomass (g) of seeds produced at each transect. We identified each seed to species when possible, and if not, to family.

We employed general linear models based on a negative binomial distribution [35] using the “MASS” package in Program R [36] with α = 0.05 to compare seed density and biomass between invaded and non-invaded habitats. We used this analysis because our data violated key assumptions of normality and homoscedasticity made by ANOVA/*t*-test models.

### Cheatgrass seeds and granivores

We estimated the flux of resources provided by cheatgrass seeds through communities of native rodents by comparing paired control and experimental (cheatgrass-supplemented) populations of small mammals at three independent sites. All sites were located in non-invaded shrubland (cheatgrass cover 0–5%; see [33] for protocol) and were all separated by > 5 km, a distance that exceeds the typical home range size of small mammals in our study area by ~5000% [37]. Thus, small mammal communities at each site were spatially independent. Each site consisted of two paired plots, each measuring 90 x 90 m and separated from one another by 50–100 m. We subdivided each plot into 10 x 10 trapping grids with 100 stations separated by 10 m in all directions. We used a mark-recapture technique with Sherman live traps (H.B. Sherman Traps, Tallahassee, Florida, USA) placed at each station to determine baseline small mammal abundance, species richness, and diversity (Shannon-Wiener index; [38]) prior to supplementation treatment. We conducted pre-treatment trapping sessions during the first 10 days of April and June 2010. Cheatgrass supplementation was initiated July 2, 2010 and continued through June 2011. Post-treatment trapping sessions occurred during the first 10 days of August and October 2010, and April and June 2011.

Trapping sessions lasted three nights at each site during which the 200 traps at each site (100 at each plot) were baited <1 hour before sunset with commercially available gerbil feed (Manna Pro Products LLC, Chesterfield, Missouri, USA) and checked the following morning at sunrise. Traps were closed during the day. We placed 5 g of polyfil batting in the back of each trap during sessions when overnight temperatures were expected to dip below 5°C to reduce mortality from exposure. We marked captured individuals with uniquely numbered ear tags. We divided captured species into Heteromyid (family Heteromyidae) and non-Heteromyid functional groups. The Heteromyids are primarily obligate granivores [39] and do not hibernate, remaining active and even breeding year-round [40]. Heteromyids might therefore have exhibited a stronger response to seed supplementation. Non-Heteromyids were considered facultatively granivorous because non-seed items typically constitute a substantial portion of their diets. Thus, non-Heteromyids might have exhibited a weaker response to seed supplementation.

We randomly selected one plot at each site to receive cheatgrass supplementation. We outfitted each experimental plot with 81 feeding trays (placed in the center of each cell of the trapping grid), spaced approximately 10 m apart, alternately placed either directly under the canopy of the nearest living shrub or in shrub interspace. Feeding trays consisted of aluminum casserole tins (12 cm-diameter x 6 cm-height) buried with the rims flush to the ground. We punctured three drainage holes and placed a 3 cm x 30 cm wooden ramp in the bottom of each tray to facilitate rodent access. We filled trays with approximately 100 g of cheatgrass seed (filled by volume) the first week of every month, including winter, from July 2010 through June 2011. We harvested all cheatgrass seed used for supplementation during June and July 2010, on land managed by the Bureau of Land Management in Rush Valley and Skull Valley, UT. We avoided harvesting from patches affected by visually-apparent diseases such as head smuts. Harvesting did not alter the structural integrity of the seeds; i.e. seed hulls and awns were left intact to ensure the cheatgrass we offered to small mammals conformed as closely as possible to the form typically encountered in nature.

Since monthly cheatgrass supplementation placed significant propagule pressure on experimental plots, we took measures to prevent the establishment of nascent cheatgrass populations. Each time we filled feeding trays (July 2010 through June 2011), we mechanically removed any germinated cheatgrass plants by the roots, preventing cheatgrass individuals from maturing and reproducing, and thus precluding population establishment. In addition, we returned to study sites in three subsequent years (June 2012, 2013, and 2014) to monitor cheatgrass populations and remove any persisting individuals. Fortunately, we found very few cheatgrass plants during these subsequent visits, suggesting our removal efforts adequately controlled nascent cheatgrass populations. Accordingly, we posit that our supplementation experiment has had little long-term impact on study plots.

We used a repeated-measures ANOVA in Program R [36] with α = 0.05 to elucidate the effect of supplementation on abundance, species richness, and diversity of small mammals over time relative to pre-treatment baseline data. If seed resources provided by cheatgrass invasion subsidize small mammals, abundance and diversity on experimental plots should increase over time, whether as a consequence of immigration of nearby animals or a reproductive response.

All capture and handling procedures were approved by the Brigham Young University Institutional Animal Care and Use Committee (protocol #090302). All care was taken to minimize rodent stress during handling. We captured no threatened or endangered species.

### Food preference

To examine how the food preference of native consumers might mediate the trophic integration of cheatgrass seeds, we conducted seed choice experiments during October 2010 on five– 550 m transects. All transects were located in non-invaded habitat (cheatgrass cover 0–5%; see [33] for protocol) and were spaced >10 km from one another and >1 km from any other study area used in this work. We situated three transects in non-invaded big sagebrush communities and two in non-invaded black greasewood communities. Each transect consisted of 12 stations separated by 50 m. At each station, we placed two 45 x 45 x 2 cm aluminum trays filled with 3 L of on-site soil, sieved through a 1 cm mesh. We placed trays directly on the soil surface, side by side. We designated one tray for native seeds and the other tray for cheatgrass seeds. The native tray consisted of 3 g (dried at 60°C for 12 hours) of either Indian ricegrass (hereafter “ricegrass”, *Achnatherum hymenoides*) or bottlebrush squirreltail (hereafter “squirreltail”, *Elymus elymoides*) seeds. The native species offered at each station alternated between ricegrass and squirreltail. Native seeds were donated by the Great Basin Research Station in Ephraim, UT. Similarly, cheatgrass trays contained 3 g (dried at 60°C for 12 hours) of cheatgrass seed. We raked all seeds into the soil of each tray and left trays undisturbed in the field for one week, after which we transferred the contents of each tray into paper sacks and oven-dried them for one week at 60°C. After drying, we stored samples at room temperature until analysis.

To recover seeds from the soil, we first passed each sample through a 1680 μm sieve (to remove rocks and large debris) and then a 500 μm sieve (to retain seeds and small debris) stacked on top of a solid base for 12 minutes. After sieving, we floated the seeds from the soil [41] and decanted them using the valve method [42]. We then dried all matter (including leaf and root litter and other organic debris) recovered from flotation at 60°C for 12 hours, and picked the seeds out with tweezers. We redried the recovered seeds at 60°C for 12 hours, weighed them, and subtracted this figure from 3 g to determine how much seed was removed. We assumed that preference and seed removal were positively correlated. We compared seed removal for each species across all transects using analysis of variance in Program R [36] with α = 0.05 after square root-transforming data for normality. Since preference trials were conducted in distinct plant communities (i.e., big sagebrush-dominated and black greasewood-dominated), we accounted for any effects of transect location using a mixed-models general linear model (glm) with provenance (native seeds vs. cheatgrass seeds) as a fixed factor and transect location as a random factor. This analysis was conducted with SAS statistical software (SAS Institute, Inc., Cary, NC, USA).

We suggest that seed removal in this experiment was driven primarily by small mammals. Because we raked seeds into the soil, most of the seeds in our trays were inaccessible to ants, which harvest exclusively from the soil surface and do not dig for buried seeds [43–44]. In addition, we observed no bird tracks—which are highly visible and easily distinguishable from those of small mammals—in or near seed trays at any site. However, we found many footprints from small mammals at all sites. Accordingly, we argue that although granivory from ants and birds was theoretically possible, small mammals drove our results.

### Data deposition

Our data are archived in the Dryad Digital Repository: http://dx.doi.org/10.5061/dryad.1v243.

## Results

### Cheatgrass invasion and seed resources

**Fig 1:**
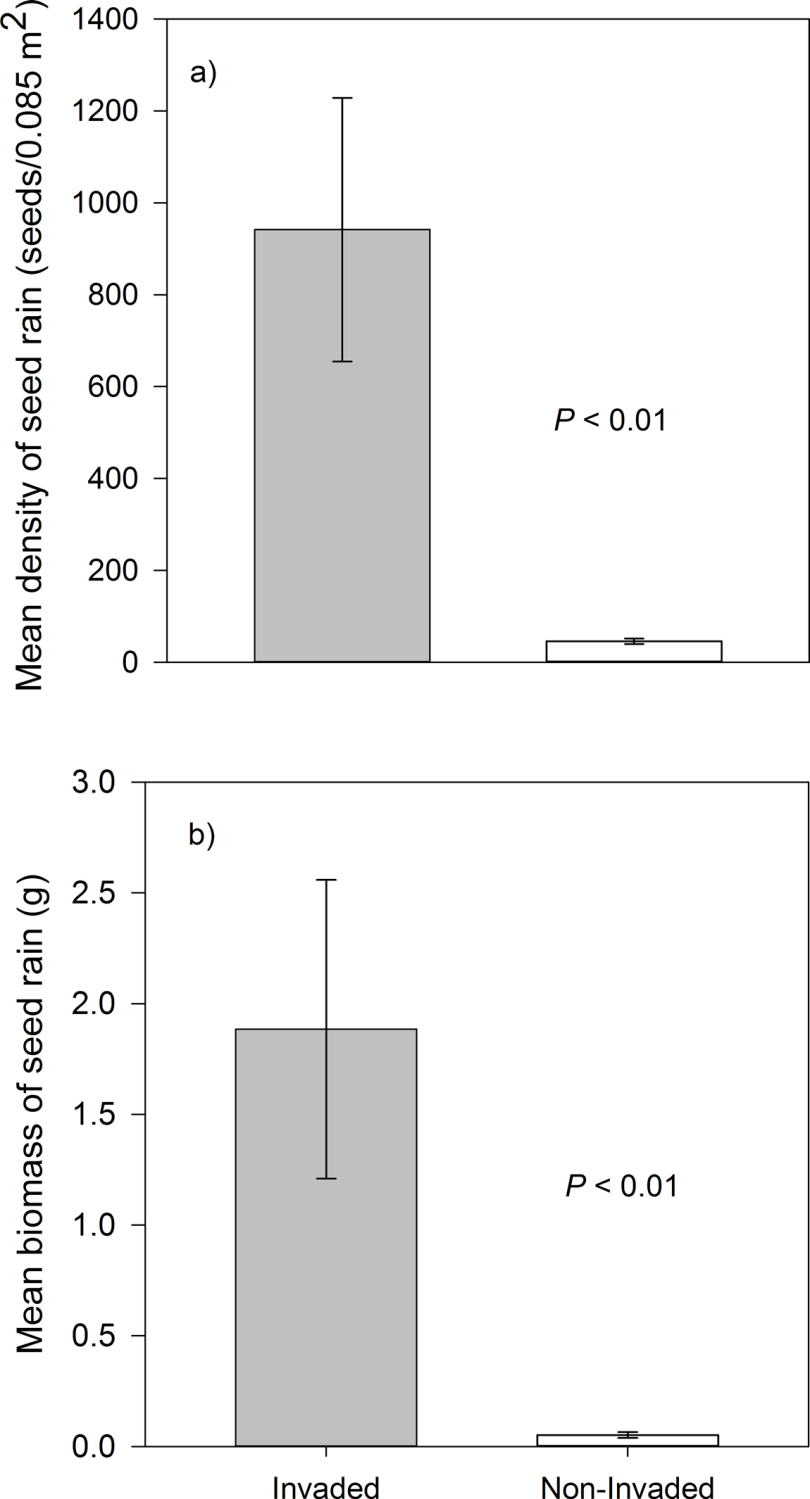
Seed Production on Cheatgrass-invaded vs. Non-invaded Habitat. Comparison of the a) density (seeds/0.085 m^2^) and b) biomass (g) of seed rain (mean ± SE) from cheatgrass-invaded (“Invaded”) and non-invaded (“Non-Invaded”) sage-steppe habitats in a Great Basin ecosystem (n = 6).

Relative to non-invaded habitat, total seed production in cheatgrass-invaded habitat was over 2000% denser (*P* < 0.01; Table 1 and Fig 1a) and over 3500% more massive (*P* < 0.01; Table 1 and Fig 1b). Not surprisingly, cheatgrass accounted for the greatest proportion of seeds produced in invaded habitat (Table 1). Interestingly, however, seed production from squirreltail, a native perennial grass, was over 1100% (*P* < 0.01) denser in cheatgrass-invaded habitat than non-invaded habitat. Seed production from Asteraceae was nearly 300% greater in non-invaded habitat (*P* = 0.02; Table 1).

**Table 1:** Seed Production on Cheatgrass-invaded vs. Non-invaded Habitat.

Seed Source	Seed Density (seeds/0.085 m^2^) Non-Invaded	Seed Density (seeds/0.085 m^2^) Invaded	% Diff	Biomass Non-Invaded (g)	Biomass Invaded (g)	% Diff
*B. tectorum*	2.33 (1.86)	650.67 (271.23)	27925.62*	0.005 (.004)	1.469 (0.671)	29380.00*
*E. elymoides*	15.33 (7.87)	181.67 (102.59)	1184.78*	0.030 (0.015)	0.332 (0.188)	1106.67*
Asteraceae	21.33 (4.70)	7.33 (3.39)	-291.00*	0.013 (0.003)	0.004 (0.002)	-325.00*
Brassicaceae	5.00	85.00 (84.00)	1700.00	0.004	0.065 (0.064)	1625.00
Chenopodiaceae	0.00 (0.00)	10.00	-----	0.000	0.011	-----
Malvaceae	0.33	0.00	-----	0.000	0.000	-----
Unknown	0.33	1.33	403.03	0.000	0.000	-----
Total	45.67 (5.78)	941.67 (287.03)	2061.90*	0.052 (0.013)	1.884 (0.674)	3623.08*

Relative contributions to the mean density (seeds/0.085m^2^) and biomass (g) of viable seeds captured in cheatgrass-invaded (“Invaded”) and non-invaded (“Non-Invaded”) shrub-steppe in a Great Basin ecosystem (± SE; statistics not reported where means = SE). Some SE are relatively large because data were pooled at the transect level (n = 6). Differences in production (% Diff) between habitat types are also reported, with statistical significance (*P* ≤ 0.05) denoted by an asterisk (*). Cheatgrass (*Bromus tectorum*; Poaceae) and bottlebrush squirreltail (*Elymus elymoides*; Poaceae) were sufficiently common to merit specific consideration.

### Cheatgrass seeds and granivores

We captured deer mice (*Peromyscus maniculatus*), Great Basin pocket mice (*Perognathus parvus*), chisel-toothed kangaroo rats (*Dipodomys microps*), Ord’s kangaroo rats (*D*. *ordii*), least chipmunks (*Tamias minimus*), house mice (*Mus musculus*), grasshopper mice (*Onychomys leucogaster*), sagebrush voles (*Lemmiscus curates*), and desert woodrats (*Neotoma lepida*) at our study sites. We classified these species into Heteromyid and non-Heteromyid groups as previously described. We captured sufficient numbers of deer mice, pocket mice, chisel-tooth kangaroo rats, and Ord’s kangaroo rats to perform species-specific analyses on their respective numeric responses to cheatgrass supplementation.

All small mammal species included in our analyses exhibited at least two generations, and most bred and recruited juveniles at several periods throughout the year, including winter months (see also [40, 45]). Notably, the most abundant small mammal encountered, *P*. *maiculatus*, has an average lifespan of < 4 months [45]. Thus our 11 months of cheatgrass supplementation encompassed multiple consumer generations and was an appropriate interval in which to detect community-level numeric responses to dietary supplementation [46]. Importantly, most food supplementation experiments involving small mammals in field conditions report significant responses from target communities in <1 year [46].

**Fig 2:**
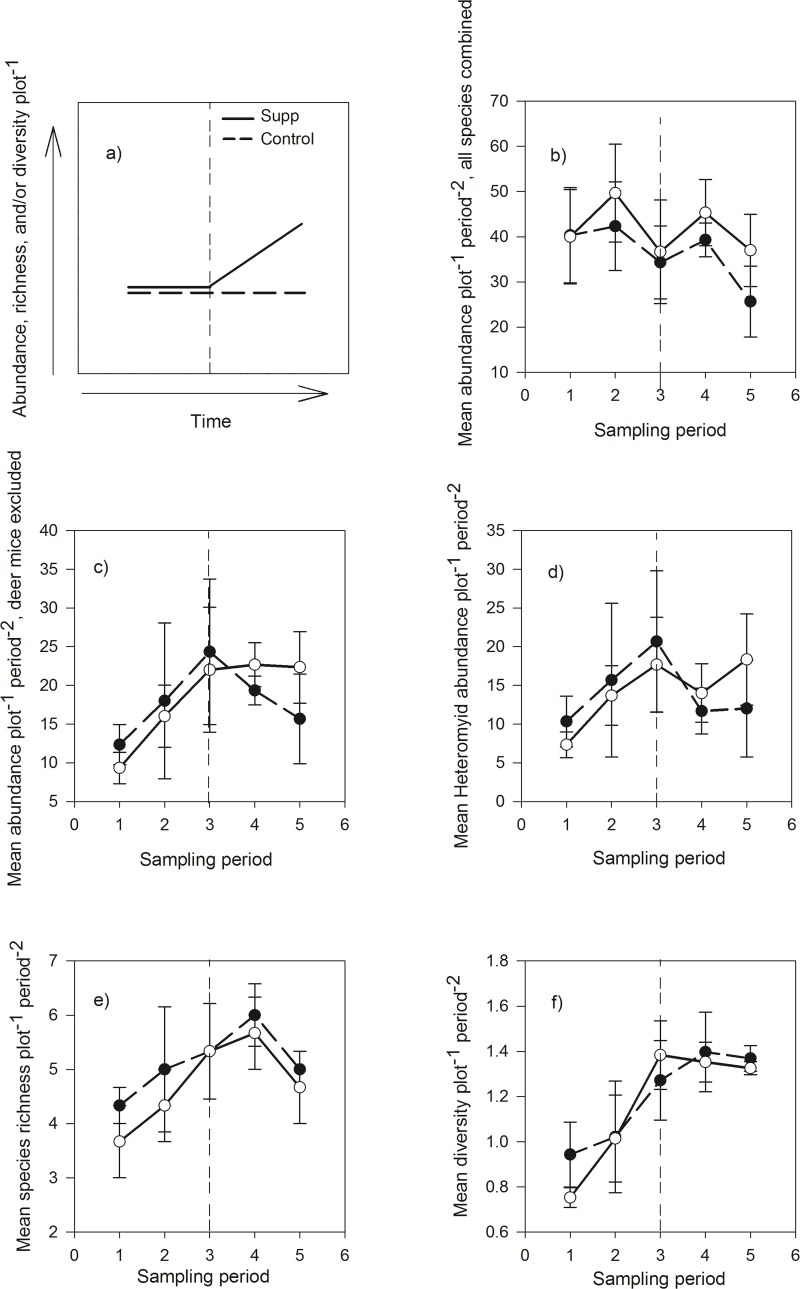
Effects of Cheatgrass Supplementation on Small Mammal Communities. Graph a) depicts the predicted effect of cheatgrass supplementation on the abundance, species richness, and/or diversity of native consumers over time. If additional resources subsidize native consumers, supplementation should cause changes in consumer demography over time (line slope) to diverge between control (“Control”) and cheatgrass-supplemented (“Supp”) communities. Graphs b-f) depict the observed effect of cheatgrass supplementation over time (“Sampling period”) on mean ± SE b) abundance of all small mammals combined, c) abundance of all small mammals combined excluding deer mice, d) abundance of Heteromyids, e) species richness, and f) Shannon-Wiener diversity on control (non-supplemented; filled circles) and experimental (cheatgrass-supplemented; open circles) plots in a Great Basin ecosystem (n = 3). Vertical dashed lines represent the time at which cheatgrass supplementation was initiated. All *P* > 0.55.

As a whole, small mammals showed no numerical response to cheatgrass supplementation (*P* = 0.79; Fig 2b). This result could have been driven by deer mice, which were over three times more abundant than any other species at our study sites. To address this, we performed a separate analysis with deer mice excluded, but our results were unaffected (*P* = 0.76; Fig 2c). Thus, deer mice alone did not drive our results. In addition, neither Heteromyids as a whole (*P* = 0.78; Fig 2d) nor any individual species of Heteromyid numerically increased in response to cheatgrass supplementation (*P* = 0.59 for pocket mice, 0.92 for Ord’s kangaroo rats, and 0.32 for chisel-toothed kangaroo rats). Facultative (i.e., non-Heteromyid) granivores were similarly unaffected (*P* = 0.99). Cheatgrass supplementation did not affect the species richness (*P* = 0.97; Fig 2e) or diversity (*P* = 0.94; Fig 2f) of rodent communities.

Although we observed evidence of small mammals interacting with seed trays, we did not actually observe utilization of seed resources. During winter months, we often observed Heteromyid tracks in the snow apparently leading from one feeding tray to the next (kangaroo rats leave very distinctive footprints; see [47]). We therefore suppose that feeding trays were visited at least occasionally by Heteromyids. However, we never observed appreciable depletion of supplemental seeds. In fact, it often appeared that no seed whatsoever had been removed from any feeding tray, even during winter months.

### Food preference

**Fig 3:**
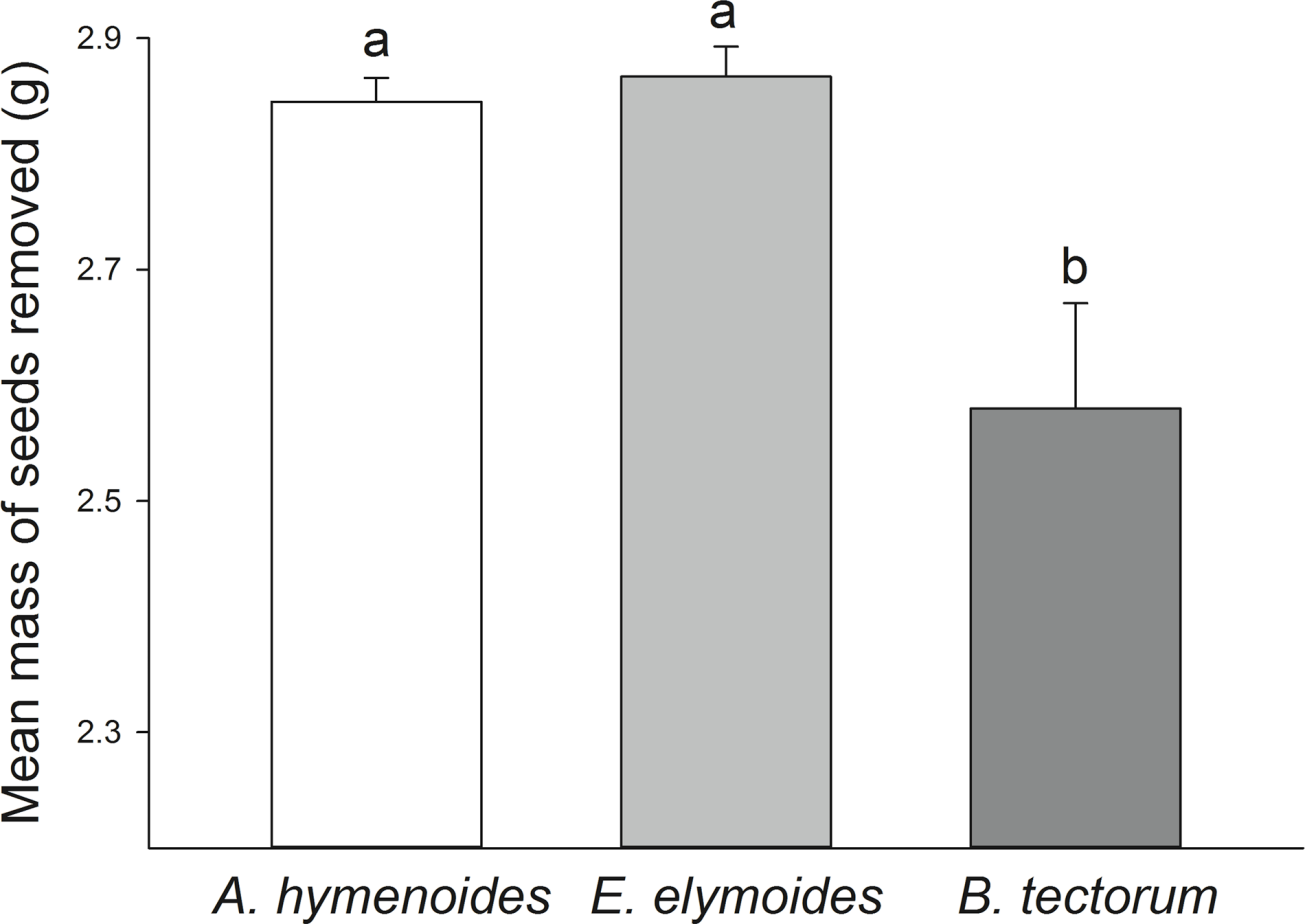
Seed Preference of Native Granivores. Mean ± SE biomass (g) of seeds of ricegrass (*Achnatherum hymenoides*), bottlebrush squirreltail (*Elymus elymoides*), and cheatgrass (*Bromus tectorum*) removed by native granivores during seed preference trials in a Great Basin ecosystem (n = 5). Means with same letter do not significantly differ at *P* = 0.05.

Granivores more heavily depredated native seeds than cheatgrass seeds, although they did not discriminate between native species. For all transects combined, granivores removed nearly identical masses (*P* = 0.91) of ricegrass (2.85 g ± 0.03 SE) and squirreltail (2.87 g ± 0.04 SE). By comparison, seed predators removed significantly less (*P* < 0.01) cheatgrass seeds (2.58 g ± 0.09 SE) (Fig 3). Mixed-models glm analysis confirmed that native granivores removed more native seeds than cheatgrass seeds (*P* < 0.05) and, importantly, revealed no significant effect of transect location (*P* = 0.06). In addition, we found no interaction between transect location and provenance (*P* = 0.09). Thus, native granivores preferred native seeds over cheatgrass seeds at all sites with no evidence of context dependence.

## Discussion

Our results show that cheatgrass invasion dramatically increased ecosystem-scale seed production (Fig 1), an important component of net primary productivity. This result coincides with many other studies linking plant invasions to increased productivity [1–2]. It is interesting to note that while invasion generally increases plant production by about 85% [1–2], cheatgrass invasion in this study increased seed production by over 3500% (Fig 1). Cheatgrass invasion thus markedly enhanced the quantity of seed resources available to native granivores.

However, we found no evidence that experimental additions of cheatgrass seeds subsidized the abundance or diversity of granivorous small mammals (Fig 2). This lack of a response suggests that seed resources provided by cheatgrass may not rapidly flux to native rodents and is consistent with many studies that link cheatgrass dominance to depauperate small mammal communities in the Great Basin [30–32, 48]. There are many examples of exotic species altering native food webs (e.g., [5, 49]), but to our knowledge, this is the first field study to experimentally follow the flux of food resources from an invasive plant to a higher trophic level in a native, terrestrial food web.

Our preference trials suggest a potential mechanism for why cheatgrass supplementation failed to enhance small mammal abundances. Native granivores disproportionately rejected cheatgrass seeds in favor of native seeds (Fig 3). Our findings coincide with other reports that Great Basin granivores prefer native seeds over cheatgrass [25, 28–29]. Thus, the notion that native consumers generally prefer exotic plants [22] appears to be unsupported in this system. This is not to say that native granivores cannot or will not eat cheatgrass seeds under all conditions. Small mammals in the Great Basin are morphologically and physiologically capable of extracting calories from cheatgrass seeds [50], and cheatgrass seeds often constitute a portion of their diets [19–21, 50]. Indeed, native granivores removed a substantial proportion of cheatgrass seeds offered in our preference experiments (Fig 3). Therefore, our results should be explicitly interpreted in the context of *preference* for native seeds over cheatgrass.

Many authors have shown that North American granivores avoid cheatgrass seeds [25, 28–29], but the reasons why are unclear. Seed preference could reflect a number of seed attributes including size, caloric value, mineral nutrition, and physical and chemical defenses. Cheatgrass seeds in our preference experiments were smaller than either native species (cheatgrass: 2.54g/1000 seeds; ricegrass: 3.53g/1000 seeds; squirreltail: 3.50g/1000 seeds), but seed mass does not always predict preference [6, 25]. Alternatively, granivores could select against cheatgrass seeds because of relatively poor mineral nutrition (although caloric value seems comparable to several more-preferred species [25, 51]). In addition, cheatgrass seeds possess relatively long awns that could deter granivory [25], but many native seeds—including squirreltail used in our experiments—also possess long awns. It is therefore unlikely that awns alone accounted for our results. Finally, cheatgrass seeds may be chemically defended by yet unrecognized compounds. Such compounds may include the by-products of biotic agents inhabiting the seeds, including fungal pathogens (e.g. *Pyrenophora* [16]) and/or endophytes (e.g. *Morchella* [52]). Interestingly, some native granivores avoid seeds infected by endophytic and saprophytic fungi [44].

Other factors besides seed preference may have influenced the failure of cheatgrass supplementation to subsidize small mammals in this study. It is important to note that small mammals may not have been food limited at the temporal and spatial scale of our study. Precipitation is a crucial determinant of yearly seed productivity in arid environments like the Great Basin [39], and granivores seem to forage primarily from the seed production of the current year [34]. Given that precipitation during the supplementation period was approximately 35% greater than the area’s 30 year average (27.3 cm year^-1^ [53]), our sites probably experienced above-average seed productivity. If so, the availability of preferred seeds may not have dwindled sufficiently to induce appreciable consumption of cheatgrass seeds. This scenario is consistent with dietary models predicting that non-preferred food items should be ignored even when superabundant as long as preferred items persist [54]. In other words, small mammals may have little incentive to consume seeds from less-preferred species like cheatgrass until seeds from preferred sources become scarce. Perhaps in dry years small mammals could more readily turn to less-preferred species like cheatgrass [45–46].

Furthermore, supplementation may not have subsidized small mammals if the study period was too brief to elicit reproductive and/or aggregative responses from target communities. We argue that this was not the case, however. Since most species of small mammals at our study plots bred and recruited juveniles several times during the supplementation period, additional resources from cheatgrass seeds had the potential to influence the survival and fecundity of multiple generations. Thus, the supplementation period was adequate to elicit a reproductive response from target communities. In addition, cheatgrass supplementation had the potential to subsidize small mammal populations via immigration from adjacent habitats. Such immigration occurs over short temporal scales within the lifetime of individuals; a period of < 4 months for deer mice [45]. Thus, our 11-month supplementation period was also sufficient to elicit aggregative responses via immigration. These conclusions are consistent with Boutin’s [46] review of 138 field experiments of dietary supplementation. Boutin noted that supplementation periods of < 1 yr. generally resulted in two- to three-fold increases in the density of target populations of terrestrial vertebrates. Importantly, however, positive responses to food supplementation are only predicted if the value of supplemented patches exceeds the value of unsupplemented patches [55–57]. This may not be the case if supplemental resources are inferior to those from unsupplemented plots [55–56].

Finally, it is possible that small mammals at our study plots were unable to locate trays of supplemental cheatgrass seeds. However, field observations do not support this idea. We routinely observed Heteromyid tracks apparently leading from one feeding tray to the next at supplemented plots, suggesting that small mammals successfully located and visited seed trays. Despite visitation, however, it often appeared that no seeds whatsoever had been removed from any supplemental trays. Together, these observations suggest that resident small mammals located and investigated supplemental seed trays, but generally refused their contents.

Why, then, should small mammals remove appreciable amounts of cheatgrass seeds in preference trials but apparently ignore them in supplementation experiments? A plausible explanation involves the effects of divergent seed neighborhoods in the preference vs. supplementation experiments. Both theoretical [56–57] and empirical [58–59] work suggests that the identity of a plant’s neighbors can strongly influence whether it experiences herbivory. For example, fall cankerworms (*Alsophila pometaria*) are generalists that attack box elder (*Acer negundo)* and poplar (*Populus spp*.) trees, although cankerworms strongly prefer box elders to poplars. However, relatively unpalatable poplars experienced significantly higher rates of cankerworm attack when growing under the canopies of palatable box elders than when growing alone or near conspecifics [60]. Increased risk of predation via proximity to palatable neighbors is called “associational susceptibility,” and is widely-reported in plant-herbivore systems [59]. Since cheatgrass seeds were always paired with those of more-palatable neighbors (i.e., ricegrass or squirreltail) in our preference trials, associational susceptibility may have inflated rates of cheatgrass removal relative to supplementation experiments, where cheatgrass seeds were never paired with palatable neighbors.

It is tempting but inappropriate to interpret the results of our supplementation experiments in the context of apparent competition. Apparent competition occurs when an increase in alternative prey increases the local density of generalist predators, which intensify consumption on nearby focal prey [57, 61]. Thus, apparent competition is a negative indirect interaction between prey species driven by shared predation. Our experiments provide data on aspects of this scenario, but are insufficient to definitively infer apparent competition. We provide strong evidence that cheatgrass invasion increased the availability of alternative prey—cheatgrass seeds, and we investigated whether this increase could augment populations of polyphagous predators—small mammals. However, we collected no data on the *effect* of cheatgrass seeds on native seeds through shared predation, making apparent competition impossible to verify. Several recent studies have argued that apparent competition can exacerbate plant invasions [62–64], and some evidence suggests that shared predation could contribute to cheatgrass invasion in the Great Basin [17], but the importance of apparent competition per se remains unknown in this system. However, we note that by definition apparent competition is unlikely when alternative prey fails to enhance local densities of generalist consumers [57–58, 61].

The higher number of squirreltail seeds produced in cheatgrass-invaded plots was remarkable (Table 1), but corresponds with studies showing that established *Elymus spp*. are strong competitors against cheatgrass and may resist local extirpation in native communities [65–66]. Since seed inputs from other native species are often reduced to negligible quantities in cheatgrass-dominated habitat [67], it is striking that seed production from squirreltail should not only persist, but increase with cheatgrass dominance. This suggests an interesting but untested possibility that cheatgrass may suppress other native competitors that in turn suppress squirreltail, indirectly releasing this native.

In conclusion, our data suggest that cheatgrass invasion dramatically increased ecosystem-scale seed production (Table 1 and Fig 1), but this production did not subsidize the abundance or diversity of granivorous small mammals (Fig 2), potentially because native consumers distinctly preferred native seeds over cheatgrass seeds (Fig 3). Thus increased primary productivity from a dominant invader may not appreciably flux through native trophic levels. In other words, plant invasions may subsidize primary productivity without subsidizing native consumers. In this context, cheatgrass invasion could disrupt native food webs by providing less-preferred resources that fail to enrich higher trophic levels. Perhaps the failure of native consumers to harvest exotic biomass helps explain the relatively inflated net primary productivity of many invaded ecosystems [1–2].
